# Micro/Nano Technologies for High-Density Retinal Implant

**DOI:** 10.3390/mi10060419

**Published:** 2019-06-22

**Authors:** Qi Zeng, Saisai Zhao, Hangao Yang, Yi Zhang, Tianzhun Wu

**Affiliations:** 1 Shenzhen Institutes of Advanced Technology (SIAT), Chinese Academy of Sciences (CAS), Shenzhen 518055, China; qi.zeng@siat.ac.cn (Q.Z.); ss.zhao@siat.ac.cn (S.Z.); hg.yang@siat.ac.cn (H.Y.); 2 Shenzhen CAS-Envision Medical Technology Co. Ltd., Shenzhen 518100, China; zhangyi@cas-envision.com

**Keywords:** retinal implant, high-density, implantable packaging, microelectrode array, coating

## Abstract

During the past decades, there have been leaps in the development of micro/nano retinal implant technologies, which is one of the emerging applications in neural interfaces to restore vision. However, higher feedthroughs within a limited space are needed for more complex electronic systems and precise neural modulations. Active implantable medical electronics are required to have good electrical and mechanical properties, such as being small, light, and biocompatible, and with low power consumption and minimal immunological reactions during long-term implantation. For this purpose, high-density implantable packaging and flexible microelectrode arrays (fMEAs) as well as high-performance coating materials for retinal stimulation are crucial to achieve high resolution. In this review, we mainly focus on the considerations of the high-feedthrough encapsulation of implantable biomedical components to prolong working life, and fMEAs for different implant sites to deliver electrical stimulation to targeted retinal neuron cells. In addition, the functional electrode materials to achieve superior stimulation efficiency are also reviewed. The existing challenge and future research directions of micro/nano technologies for retinal implant are briefly discussed at the end of the review.

## 1. Introduction

Implanted microelectronic device has enormous applications in biomedical and animal studies, and one of the emerging applications is the neural interface micro-devices for neural activity monitoring and modulation [1,2,3]. Since the first pacemaker implantation in 1958, many implantable medical devices such as deep brain stimulators, cochlear implants, and blood pressure sensors have been developed and commercialized [4,5]. These implantable medical devices typically have only 4–26 feedthroughs (or independently controlled channels), which connect the electrode array and receiver coil to the stimulator. However, higher feedthroughs within a limited space are needed for supporting more complex electronic systems and precise neural modulations. One good example is retinal prostheses to restore the basic visual functions for patients suffering from severe retinal degeneration disease including retinal pigmentosa (RP) and dry age-related macular degeneration (AMD), also known as the bionic eye, retinal prosthesis, or visual prosthesis [6,7]. AMD occurs predominately in the aged, affecting 30–50 million people globally and more than 4 million in China alone, while RP afflicts mainly children and young adults, affecting 1.5 million people globally [7,8,9,10].

**Figure 1:**
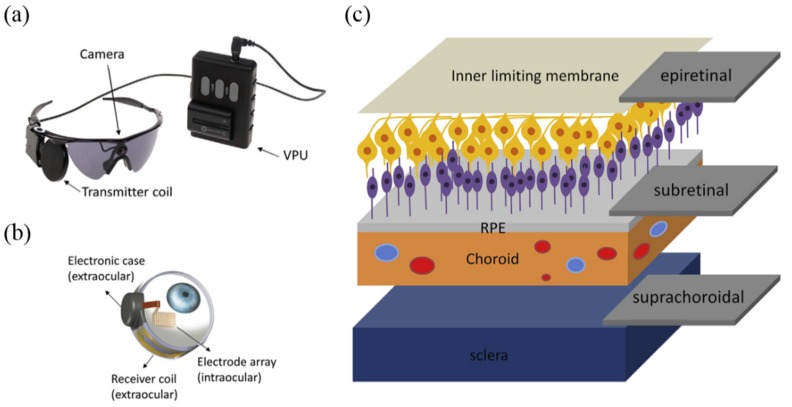
(**a**) External and (**b**) implant part of the Argus II system [7]; (**c**) illustration of the implantation sites of the visual cortex, epiretinal, subretinal, and supra-choroidal prostheses [7]. Reproduced with permission from [7], published by Elsevier, 2016.

With rapid advancements in technology and engineering for retinal implant, the first commercial retinal prostheses, the Argus^®^ II retinal prosthesis system (referred to as simply Argus II) with 60 channels from Second Sight Medical Products Inc., has obtained the European Union approval (CE Mark) in 2011, and the U.S. Food and Drug Administration (FDA) with the humanitarian device exemption in 2013, has been commercially implanted in hundreds of patients [8,9]. The working principle and clinical results of Argus II have been detailed in many review papers [7,10,11,12], and its illustration is shown in Figure 1a,b. In 2013, Alpha IMS from Retina Implant AG became the second CE certified wireless retina implant in Europe, featured as a subretinal device implanted under the damaged retina to take advantage of the signal processing capabilities of intact bipolar cells in the retina [13]. The implant uses a 3-mm square microphotodiode array (MPDA) chip consisting of 1500 photodiodes to amplify pixels instead of the eye’s photoreceptors. Another method with the retinal implantation site between the sclera and choroid, namely suprachoroid implant, was also adopted with relatively simple and less invasive surgery [14,15]. Figure 1c shows the major approaches to retinal implants depending on the implantation sites. The intraretinal neurons largely retain the ability to transmit signals despite reorganization and cell loss. The images captured from the visual field through the image acquisition device are translated into stimulation patterns for the electrode array placed near the retina. An electrochemical interface can be formed with physiological saline. The current delivered into the extracellular region causes charge redistribution on the membrane of retinal neurons, and an action potential is triggered when the membrane depolarization exceeds a threshold. For example, a negative charge accumulates outside the membrane under the electrode during cathodic stimulation, driving the intracellular positive charge moving to the electrode/neuron interface from the adjacent compartments, and leading to a strong membrane depolarization closer to the electrode and a weaker hyperpolarization far away. Anodic stimulation is the opposite [7,16]. Besides RP patients, more than 600 AMD patients received an entirely different type of implant to restore their poor vision: the implantable micro-telescope (IMT) from Vision Care of Saratoga, California [17]. The pea-sized IMT is implanted behind the iris of eye. The loss of central vision can be magnified 2.7 times by the telescope and projected onto the healthy part of the retina surrounding the scarring.

There is a long-lasting effort to increase the resolution and visual acuity of retinal implant [6,18,19]. Previous simulation has shown that a super-high density of 600–1000 stimulus electrodes (pixels) is required to help blind people with face recognition and reading large text in newspapers [20]. For this purpose, high-density implantable packaging and flexible micro electrode array (fMEA) as well as high-performance coating materials for stimulation are crucial to achieve high resolution, which will be the focus in this review.

Considering the security issues of multilayer in chronic use, all implants must be stable, biocompatible, and ensure minimal immunological reactions [21,22,23]. Most active implant devices use silicon (Si)-based electronic devices to perform the functions of sensing or treatment. Due to the biological incompatibility of Si substrate, higher density input/output (I/O) pins and peripheral circuit communication are required. There is a great demand in shrinking the implant size while increasing feedthrough counts [24,25,26,27], which has brought great challenges for packaging. Conventional direct packaging using plastic, metal, or ceramic cannot guarantee the long-term moisture-free environment for high-channel-count electronic devices. When water vapor inside the packaging exceeds a threshold value, dewing may occur, and the water droplets together with residual ions will form an electrolytic environment, leading to corrosion, delamination, cracking, or other degradations of the internal components. Therefore, electronic packaging with high density, air tightness, and biocompatibility is an inevitable choice, to build a seal cavity with good mechanical performance and biocompatibility [28,29]. 

For precise neuronal stimulation, smaller and more sophisticated microelectrode arrays are of increasing interests [30,31]. Considering the major target cells for stimulation, retinal ganglion cell (RGC) has the soma size in 5–20 μm range, so the ideal electrode size should be similar to this size to facilitate one-on-one stimulation. Actually, the optimal size of electrode suggested by the electrophysiologic experiments should be 10 to 20 μm, and the spacing between 20 and 60 μm [32], which are far less than those in current commercial products (for example, the electrodes of the Argus II are 200 μm in diameter and 300 μm in spacing). The decreasing size of electrodes will increase the impedance and improve the signal-to-noise ratio (SNR) dramatically. Moreover, the fabrication process of high-density fMEA in limited size is challenging because of low yield and easy delamination. Therefore, it is critical to study the electrode fabrication process, materials, and surface modification. 

For those tiny microelectrodes, high capacitance and charge injection capability (CIC), low electrical impedance, as well as the biocompatibility, are of great importance to reduce power consumption, lower heat dissipation, and ensure safe stimulation [33,34]. Many researchers have attempted to increase the effective surface area of microelectrodes of the given size to improve the charge delivery characteristics [35,36]. A fundamental consideration for the retinal prosthesis is the selection of proper electrode materials which should exhibit biocompatibility and be able to deliver sufficient charge within safe limits. The requirement of porous and rough coatings on the bare microelectrodes has brought opportunities for the application of various nanomaterials in the retinal implant.

It is worth to note high-density stimulation is not equal to high visual acuity (resolution), which requires a lot of factors in both hardware (electrodes and coatings) and software (stimulation strategies based on surgical results). For example, Argus II with 60 channels could only restore the patient’s visual acuity to 200/1262 without amplification [37], and Alpha-IMS with 1500 photoelectric pixels had, at its best report, up to 20/546 [38], only slightly higher than Argus II and far below the expected visual acuity. Here we will summarize only the recent progress in the device’s fabrication towards high-resolution retinal implant. The survey of stimulation strategies for retinal prosthesis can be found in other reviews [7,39].

## 2. Packaging and Integration

The packaging of implantable biomedical components has been one of the greatest challenges for chronic implantation. All the materials exposed must be highly biocompatible, and also highly inert to the erosion of body fluids. Now there are two typical kinds of packaging approaches: hard packaging and soft packaging. The former has a hard shell or capsule made of metal, glass, or ceramics, which allows multiple connections to the components of the basement or integrated into the system of internal packages. It has been widely used in commercial products and allows mass production [40], but the manufacturing costs and risk of failure are rapidly increasing for high-density feedthroughs. Many efforts have been dedicated to increase package feedthrough and reliable bonding/joint using new processes and materials [26,27,28,29]. The second option is using one or several layers of biocompatible soft films as a hermetical coating, which has the advantages of being a small-size, light-weight, low-cost, and simple process with high flexibility [41], and has attracted a lot of research interest as an emerging technology enabling high-density, ultra-small medical implants.

### 2.1. Hard Packaging and Integration

**Figure 2:**
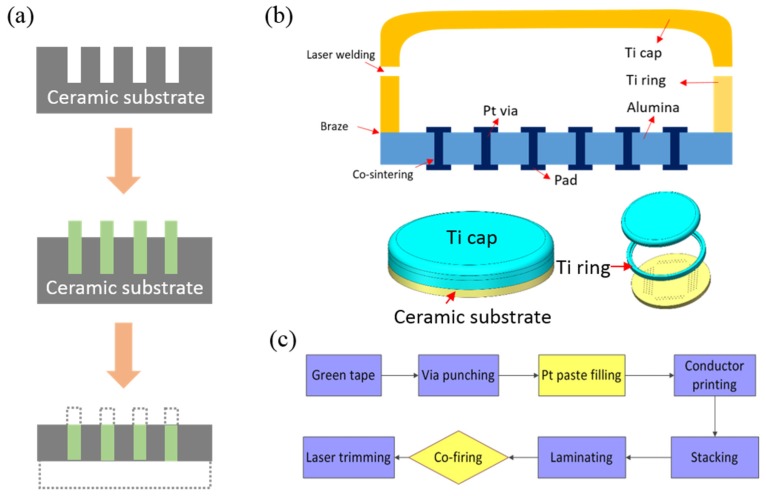
(**a**) Illustration of the package fabrication process described by a patent from Second Sight; (**b**) the cross-sectional (top) and 3D illustration (bottom) of implantable body for retinal implant with 100+ channel, and (**c**) the major process flow for platinum/alumina composite substrate in our group.

A typical hard packaging for retinal implant is the ceramic/metal composite can for Argus II. The implantable packaging includes three steps: (a) the fabrication of ceramic substrate with Pt feedthroughs, (b) achieving brazed joint with a titanium (Ti) ring, and (c) laser welding with a Ti cap. The illustration of a patented method for the fabrication process is shown in Figure 2a [42]. Multiple blind holes inside a green, or unfired, ceramic sheet were formed firstly, solid wires (or pins) like platinum (Pt) were inserted, followed by sintering treatment for the shrinkage of ceramic and removing the extra materials, so that the lower ends of the wires were flush with the lower surface of the finished sheet. 

Our group in Shenzhen Institutes of Advanced Technology (SIAT), Chinese Academy of Sciences (CAS) also adapted the hard packaging approach to host the retinal implant with more than 130 feedthroughs, but the microfabrication process was optimized in a different way. The packaging mainly consists of alumina/platinum (Al_2_O_3_/Pt) substrate, a Ti ring, and a Ti cap, which allows 100–500 feedthroughs in 1 cm^2^ and shows the best cytotoxicity grade (Grade 0) [43]. As shown in Figure 2b, Pt vias were embedded in Al_2_O_3_ sheets and covered with Pt pads for electric connection. The Ti ring was brazed with Al_2_O_3_ and bonded to Ti cap using laser welding. The whole packaging body housed circuits or power inside to be isolated from gas and water penetration.

As shown in Figure 2c, high-purity Al_2_O_3_ power and its organic adhesives were mixed into emulsion and laminated into a thin green tape. Then mechanical milling was conducted to make micrometer holes in the green tape. Pt paste was filled into the holes typically using thick-film printing technology. After that, Pt or gold (Au) line trace and pad can be patterned on the tap using screen printing technology with the aid of a stainless-steel mesh. Several patterned green tapes were aligned and stacked with uniform pressure using the commercially available isopressing equipment, followed by co-sintering of metal paste and Al_2_O_3_. Finally, the substrate was diced into desired pieces by laser cutting. The Ti ring was then brazed with Al_2_O_3_ and inserted between the Ti ring and the ceramic substrate with the aid of a thin braze alloy sheet. After mounting the integrated circuit (IC) and discrete components using surface mounted technology (SMT), the Ti ring was sealed with Ti cap using laser welding. Picosecond laser is used since it allows metal melting immediately at low-temperatures (<100 °C) to fulfill the metal interface, without additional intermediate layer. All the joint parts have reached an ultra-low level of 10^−10^ Pa·m^3^/s for helium (He), which is at least 10 times lower than the 10-year implant requirement of FDA standard (10^−9^ Pa·m^3^/s), and they also exhibit excellent biocompatibility (Grade 0) using L929 cell line.

**Figure 3:**
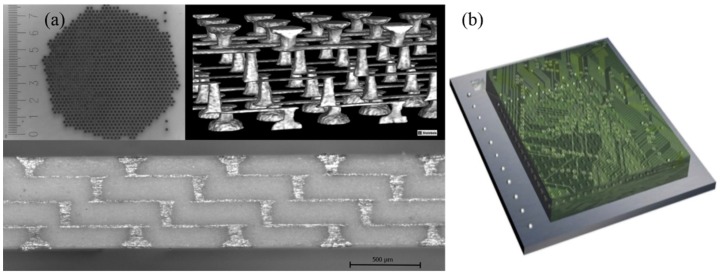
(**a**) The high-density electrode array and corresponding cross-section of hermetic feedthroughs produced with four layers, Reproduced with permission from [44], published by Wiley Online Library, 2014; (**b**) illustration of a high-density array of diamond feedthrough and electrode with 256 channels, Reproduced with permission from [25], published by Elsevier, 2014.

Another report on high-density ceramic/Pt ceramic packaging with integrated electrodes is from the University of New South Wales [44,45], up to 2500 channels per cm^2^ (Figure 3a). It is featured with chip-scale packages with integrated electronics, and the outer layer of microelectrodes can directly interface with neural tissues. The charge injection limit of these electrodes and stability under stimulation were explored, showing excellent stability under stimulation with more than 1.8 billion pulses and the electrodes have improved charge transfer properties when compared to machined Pt microelectrodes. 

There are some other materials available for the substrate, including diamond and glass. Figure 3b shows an illustration of a high-density array of diamond feedthroughs and electrodes reported by the Bionic Vision Australia (BVA) group in the University of Melbourne [25]. The 256-channel feedthrough array was constructed from a kind of polycrystalline diamond with electrically insulating diamond substrate, containing many electrically conducting nitrogen doped ultra nano-crystalline diamond (N-UNCD) feedthroughs. N-UNCD has appropriate electrochemical characteristics to act as a neural stimulation material [26,27]. Although the growth and microfabrication of diamond materials was quite complicated and expensive, this method has several advantages. First, diamond can minimize the possibility of feedthrough failure by materials mismatch, which increases the reliability. Also, diamonds exhibit excellent stability, good biocompatibility [28,42,46], and superb biochemical stability [25], offering the prospect of a long-lasting implant. 

**Figure 4:**
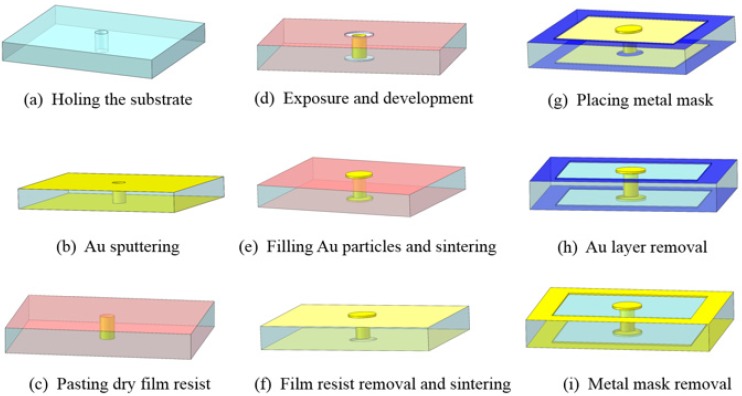
Fabrication process of the base wafer: (**a**) Holing the substrate, (**b**) Au sputtering, (**c**) pasting dry film resist, (**d**) exposure and development, (**e**) filling Au particles and sintering, (**f**) film resist removal and sintering, (**g**) placing metal mask, (**h**) Au layer removal, and (**i**) metal mask removal. Reproduced with permission from [46], published by IOPScience, 2016.

Recently, through glass via (TGV) technology was reported from Waseda University as shown in Figure 4. Similar to through silicon via (TSV) technology, TGV has several advantages, including high wiring density, lower energy consumption, and fast signal speed, compared with conventional wire bonding methods [46]. Air leaks from the TGV area are minimized by sandwiching the TGV between two Au bumps fabricated simultaneously with the TGV by a simple filling process, therefore the throughput is high compared with conventional through via technologies.

### 2.2. Soft Packaging and Integration

**Figure 5:**
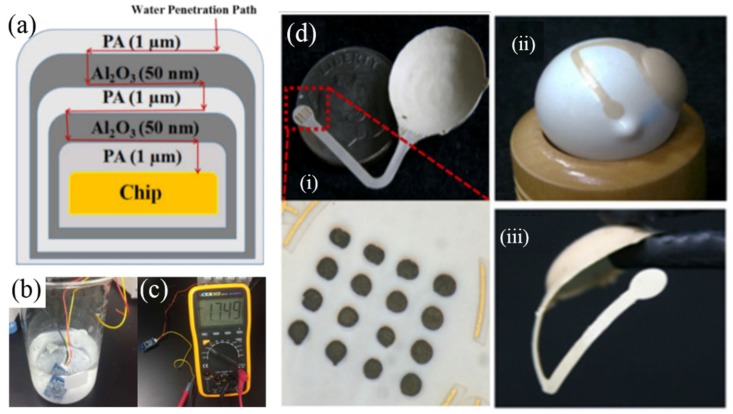
(**a**) Schematic diagram of the five-layer PA/Al_2_O_3_/PA/ Al_2_O_3_/PA film on a sensor IC; (**b**) active soaking test for the film-coated humidity sensor at 87 °C; (**c**) measurement setting of the humidity sensor after active soaking test; (**d**) fabricated LCP-based retinal prosthesis: (i) comparison with a dime and the inner surface, and magnification of the retinal electrode array coated by iridium oxide, (ii) the device on a model eye showing conformal attachment, (iii) electrode part was precurved to fit the eye-curvature. Reproduced with permission from [55], published by IEEE, 2015.

Soft packaging for medical implants borrowed the idea from the film packaging used for light emitting diode (LED) and liquid crystal display (LCD). At present, the most commonly used film packaging is the combination of multi-layer film to form a packaging barrier layer, also known as Barix packaging technology [47], requiring simple preparation process and low cost. Among the available polymer materials [41], parylene-C is the most commonly used due to its wide applications and comprehensive advantages. Parylene-silicone-parylene film has been demonstrated for 256-channel retina implants [48] and is expected to work equivalently for 7 years in an accelerated test [49]. Our group used ultra-thin (3.1 µm) and biocompatible organic/inorganic composite film based on room-temperature and conformal deposition of Al_2_O_3_ and parylene C, as shown in Figure 5a [50]. The water penetration route of such film will be significantly extended by steering the penetration path to the organic/inorganic interface, so that combining only 5 layers can significantly improve the lifetime for implantation. Its leakage rate was lower than 1 × 10^−10^ Pa·m^3^/s, with 58 days of the active soaking test for a commercial humidity sensor with this composite film at 87 °C (Figure 5b,c), expected to have the life time longer than 5 years [51].

In addition, liquid crystal polymer (LCP) is a new emerging material due to its high strength, high modulus, ultra-low water absorption, and low expansion coefficient [52,53,54]. The completed LCP-based retinal prosthetic device is shown in Figure 5d. The device has a circular package accommodating the electronics with a 14 mm diameter and 1.3 mm maximum thickness for its crescent-shaped cross section, which can be conformally attached on the eyeball. The electrode part to be inserted into the retina has a thickness of 30 μm after the laser-thinning process which etched away the LCP starting from a 350 μm thickness and is precurved to fit the eye-curvature. This LCP-based retinal prosthesis weighs only 0.38 g, less than a tenth of conventional implantable devices with a metal package. Considering that the weight of an eyeball is about 5 g, this weight reduction is a significant improvement in patients’ discomfort as well as implantation stability.

## 3. Microelectrode Array for Retinal Implant

In retinal prosthesis, fMEAs are the critical interfaces between the implant system and the tissue that deliver charge-balanced electrical stimulation to targeted retinal neuron cells [56]. Depending on the implant sites, there three main three types of retinal electrode array as shown in Figure 1c, i.e., an epiretinal one on the top surface of the retina, a subretinal one between the retina and retinal pigment epithelium (RPE)/choroid, and a supra-choroidal one on the posterior scleral surface. The following sections for the three types are reviewed in detail. 

### 3.1. Epiretinal Electrodes

**Figure 6:**
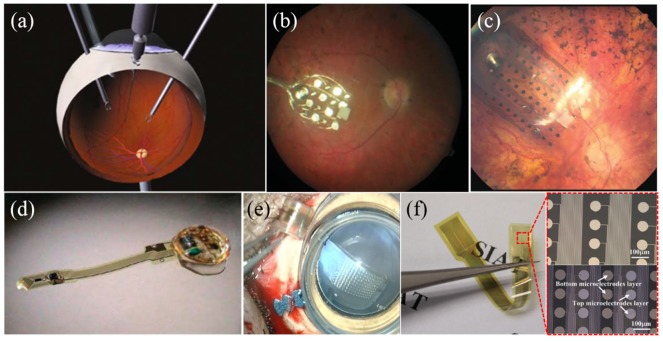
(**a**) The configuration of one of the very first patient tests [57]; (**b**) implanted 16-channel electrode array of Argus I [7]; (**c**) electrode array of Argus II implant containing 60 electrodes [66]; (**d**) an epiretinal stimulator with a thin-film polyimide cable of gold traces [59]; (**e**) 126-channel electrode implanted in the eyes of mini-pigs by our group; (**f**) 1025-channel electrode fabricated by our group [64,65]. Reproduced from the mentioned references with permission from the related journals.

Early experiments demonstrated that electrical stimulation could restore visual signals. A single needle was placed onto the retinal surface to simulate the retina by Humayun and co-workers, shown in Figure 6a [57]. The pixels increased for Argus I (16 electrodes) and Argus II (60 electrodes) (Figure 6b,c). The Argus II had been implanted in 30 patients (1 with choroideremia and 29 with RP) in the United States and Europe between 2007 and 2009, all of them could perceive light when given electrical stimulation. The implant is very expensive: about $150,000 without the surgery fee [7,58]. Another typical epiretinal implant is the 49-electrode epiretinal device from Intelligent Medical Implants (IMI) as shown in Figure 6d. Unlike Argus I and II, the power for the IMI retinal stimulator is provided through a radio frequency (RF) links, while the stimulation data could be transmitted via an infrared (IR) optical link to decouple the energy and data interference. It has been demonstrated that the implant did not cause tissue damage in the eye after implanted in 4 RP subjects for over 9 months [59]. EPIRET3 is the latest retinal model from Epi-Ret that the fits entirely within the eyeball, which eliminates the need to suture any component. It contains 25 protruding electrodes (100 μm in diameter), arranged in a hexagonal array, resulting in lower thresholds by improving contact with the retina. It was implanted in six RP patients in 2006 and all subjects reported visual sensations after implantation for 4 weeks [60]. In 2016, Pixium Vision in France commercialized a 150-electrode product (IRIS II) and obtained the CE mark [61,62]. Our group also developed the 126-electrode prototype based on polyimide (PI) substrate and Ti/Pt metal layers [63] and inserted the in the eyes of mini-pigs for 3 months with good biocompatibility (Figure 6e). We also reported fMEA of 1025 electrodes with improved adhesion and reduced impedance (Figure 6f) [64,65]. The new-generation retina implant developed by our group will be more affordable in the future. 

### 3.2. Subretinal Electrodes

**Figure 7:**
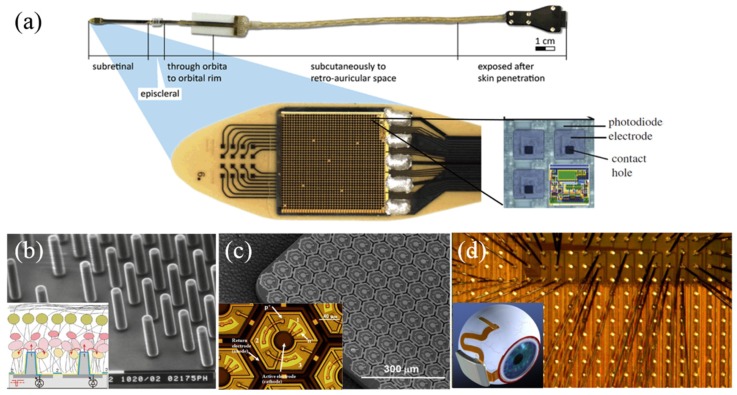
(**a**) The prototype of the Alpha-IMS predecessor, including 16 additional electrodes for direct stimulation. The microphotodiode array (MPDA) consists of 1500 photodiodes on a surface area of 3 × 3 mm [7]; (**b**) SEM of the microfabricated SU-8 pillar arrays, each pillar is about 10 μm in diameter and 40–70 μm in height. Insert: a pillar array attracts retinal cells for achieving intimate electrode-cell proximity [74]; (**c**) MPDA developed by the Palanker group. Insert: blown-up view of a single stimulating element with 3 photodiodes in series [7]; (**d**) Boston retinal implant chip, showing some of the 256 electrode current drivers. Insert: retinal implant concept with the secondary coil surrounding the cornea [73]. Reproduced from the mentioned references with permission from the related journals.

Compared to epiretinal implantation, subretinal implantation enables much higher stimulation density, however, the fMEA is highly integrated with photovoltaic circuits driven only by light inside the eyeball and has the risk of heat dissipation. There still exists disputation as to whether it could result in the atrophy around the implant area in the long term [7]. Artificial silicon retina (ASR) from Optobionics (a company from Chicago) was a typical implant of this kind with a 2-mm diameter silicon-based device, containing about 5000 microelectrodes tipped microphotodiode array (MPDA). 10 RP patients were implanted with ASR and 6 of them had vision during the 7-year follow-up observation [67]. Alpha IMS, developed by Retina Implant AG in Germany, not only used a MPDA for wireless light detection and current generation, but also included wired circuits to amplify the photocurrents to overcome the difficulty of low photovoltaic efficiency [10]. As shown in Figure 7a, the device consists of an active chip with about 1500 microphotodiodes and an additional 4 × 4 array of light insensitive titanium nitride (TiN) electrodes (50 μm × 50 μm or 100 μm × 100 μm) for direct stimulation powered externally. Six patients, after being implanted with the device, could identify simple patterns, and another one was able to recognize complex letters and forms [68,69]. 

It is vital that visual stimulation electrodes should be placed close to the target neuron cells to achieve low threshold charge and high resolution. Palanker and co-workers compared different types (flat, pillars, and chambers) of passive subretinal arrays and found that pillars had minimal alteration of the inner retinal architecture as shown in Figure 7b [70]. They developed photovoltaic retinal prosthesis adopting optical amplification instead without the need for complex electrical circuitry and trans-scleral cabling [18,71]. Each element in the photodiode array consists of a central iridium oxide (IrO_x_) electrode (Figure 7c). Photodiodes increase the dynamic range of the charge injection from the electrodes and maintain the light energy at safe levels [72]. An MIT-Harvard group developed Boston retinal implant which used a passive electrode array without MPDA for stimulation, and the new generation device had 256 electrodes as shown in Figure 7d. The earlier 15-channel prototype showed good tolerance and continued function after implanted in a mini-pig for 1 year [73]. 

### 3.3. Supra-Choroidal Electrodes

**Figure 8:**
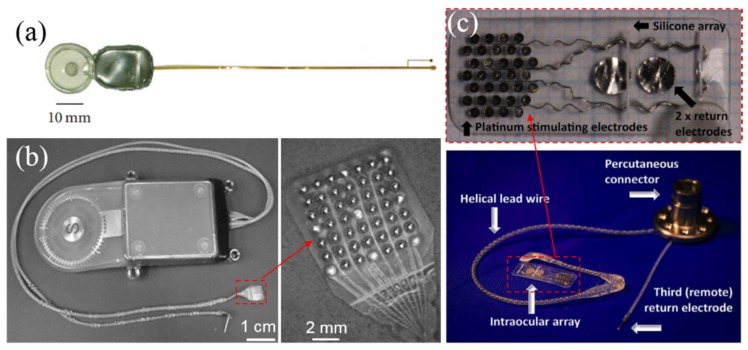
(**a**) A suprechoroidal implant for transretinal stimulation [70]; (**b**) the supra-choroidal-transretinal stimulation (STS) implant including the suprachoroidal stimulating array and the remote return electrode [77]; (**c**) the Bionic Vision Australia (BVA) implant with 1 remote return and 2 other return electrodes on the suprachoroidal array [15]. Reproduced from the mentioned references with permission from the related journals.

The electrodes for supra-choroidal implant are relatively distant from the retina leading to higher stimulation threshold [75]. Kim and co-workers from Seoul National University built a prototype implant with 7-channel electrodes as shown in Figure 8a, where the reference electrode was placed on the outer scleral surface to simplify the surgical procedures and reduce the various risks of inserting electrodes into the vitreous [76]. 

Fujikado et al. [78] developed a supra-choroidal-transretinal stimulation (STS) prosthesis as shown in Figure 8b, containing 49 Pt electrodes array with 500 μm in diameter and 700 μm separation, while only 9 electrodes were active. Nonetheless, it helped patients to localize objects better. Ayton et al. [15] from the Center for Eye Research Australia (CERA) developed another supra-choroidal prosthesis with the aim to achieve wide-view and high-resolution vision. As shown in Figure 8c, the intraocular array consists 33 Pt stimulating electrodes and 2 large return electrodes on silicone substrate. The implant remains stable during the Phase I clinical trial for 2 years [15].

## 4. Surface Modification for Electrodes

As the decreasing size of electrodes increases the impedance dramatically, it improves the signal-to-noise ratio (SNR) and reduces the stimulation efficiency in the clinical. Nanostructures and materials show great potential in improving the electrochemical and mechanical performances of neural interface in spite of the limitation size of electrodes. In the following sections, different coating materials including the key characterizations are reviewed in detail. 

### 4.1. Metallic Materials and Their Derivatives

Noble metals such as platinum (Pt), gold (Au), and iridium (Ir) are the widely applied neural electrode materials for their excellent conductivity, stability, biocompatibility, and corrosion resistance [79,80]. Pt black coating was commonly formed by electrodeposition characterized with rough structure, which could be dated back to 1894 [81,82], reducing the impedance of microelectrode at least by a factor of 4 at 1 kHz [83,84]. However, its cytotoxicity remained a serious issue due to the lead (Pb) additive in electrolyte, which was strictly forbidden in the clinic trials [80].

**Figure 9:**
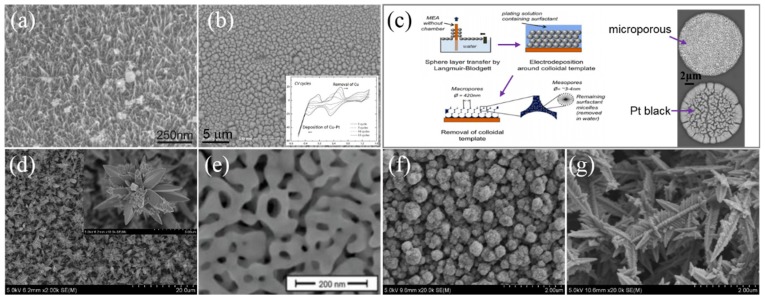
Scanning electron microscope (SEM) of different materials: (**a**) Pt-nanograss coating [80]; (**b**) cauliflower-like Pt coating [87]; (**c**) the fabrication process of depositing porous structure on electrode surface by templating [88]; (**d**) 3D nanocrystal Pt coating obtained by our group; (**e**) nanostructured Au coating by removing Ag from the Au–Ag alloy [96]; (**f**) IrO_x_/Pt nanocone composite coating in our group [94]; (**g**) IrO_x_/Pt nanoleaf composite coating in our group [95]. Reproduced from the mentioned references with permission from the related journals.

In recent years, new methods have been developed to improve the neural interfaces. Pt gray was an alternative method without cytotoxic components patented by Second Sight [35,85,86], which was electrodeposited at an intermediate rate slower than that of Pt black and possessed desirable fractal morphology and stronger mechanical strength [79,86]. Pt gray had more than sufficient chronic charge injection capacity (CIC) up to 1.0 mC⋅cm^−2^ for retinal stimulation [66], nevertheless, its impedance and cathodic charge storage capacity (CSC_c_) were still undesirable for higher density microelectrodes. To increase the electrochemical performances, Boehler et al. [80] deposited Pt-nanograss with large surface area, reducing the impedance by almost two orders of magnitude (at 1 kHz) and increasing the CSC_c_ by ~40 times compared to bare electrode, shown in Figure 9a. In addition, it also exhibited good mechanical and electrochemical stability after cleaning and pulse testing [80]. Boretius et al. [87] introduced platinum–copper (Pt–Cu) alloys and then removed Cu to fabricate a cauliflower-like micro-sized Pt, shown in Figure 9b. It exhibited good electrochemical property due to its higher effective surface area. As shown in Figure 9c, other researchers applied templating to create well-ordered and micro-structured Pt coating with good mechanical property despite of its lower electrochemical performances than Pt black [88]. Pretreatment could also facilitate the roughening of coating by reactive ion etching (RIE) before sputtering Pt on bare electrode [89]. Our group has developed various well-controlled nanocrystal Pt coatings growing on the Pt electrode using different electrodeposition methods, which are featured with strong mechanical stability and ultra-high surface area and distinguished with randomly distributed nano-aggregation. A novel three-dimensional (3D) nanocrystal Pt coating shown in Figure 9d provided extremely large surface, which significantly reduced impedance of electrode by ~93% and increased its CSC_c_ up to ~100 mC⋅cm^−2^, showing good electrochemical stability with less than 3% loss after electrical stimulation for over 2 × 10^7^ cycles [90].

Au was another promising material for neural electrode coating, the Au coating modified electrode developed by Kim et al. [91] displayed 4 times lower impedance. As shown in Figure 9e, the higher porous and interconnected Au coating obtained by co-depositing of gold–silver (Au–Ag) alloy and then removing Ag decreased the impedance by more than 25 times [92].

Despite of the advantages of Pt and Au, their CIC and CSC_c_ were still limited with the shrinking size of the electrode sites for no faradaic reaction existed [93]. Thus, materials with a higher ability to support reversible faradaic reactions such as iridium oxide (IrO_x_) were applied to promote the properties [36]. Considering the high cost and degradation/delaminate with repetitive stimulation, our group developed IrO_x_/Pt nanocone composite coating (Figure 9f) by depositing a thin IrO_x_ film on nanocone-shaped Pt to improve mechanical adhesion, demonstrating good CSC_c_ of 22.29 mC⋅cm^−2^, which was about 2.8 times higher than that of pure Pt coating [94]. And we further developed novel IrO_x_/Pt nanoleaf composite coating by optimizing the deposition condition to get higher CSC_c_ more than 400 mC⋅cm^−2^ with good stability (Figure 9g) [95], which is the best record so far.

### 4.2. Conducting Polymers 

**Figure 10:**
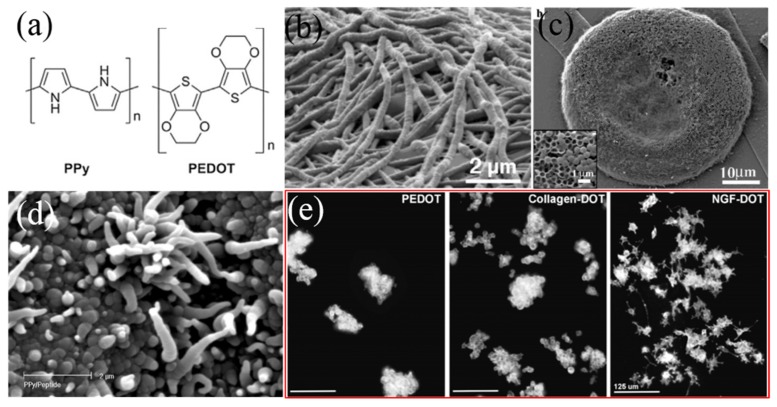
(**a**) Chemical structures of PPy and PEDOT [108]; (**b**) SEM image of PEDOT coating [109]; (**c**) SEM image of PEDOT obtained using polystyrene as template [105]; (**d**) SEM image of PEDOT/DCDPGYIGSR composite coating [106]; (**e**) Neurotrophic factor combined with PEDOT promoted the growth of neural cells [107]. Reproduced from the mentioned references with permission from the related journals.

Conducting polymers (CPs) are suitable candidate coating for neural interface which have been widely investigated, offering porous surfaces and pseudocapacitance, resulting in high CSC_c_ and low impedance [97]. In recent years, most researches have been focused on poly (3,4-ethylenedioxythiophene) (PEDOT) for its higher conductivity and biocompatibility compared to other CPs such as polypyrrole (PPy) [98]. PEDOT had higher electroactivity and stability owing to its dioxyethylene bridging group that facilitated the charge transmit (Figure 10a) [99]. It had better electrochemical stability after stimulating with charge density up to 3 mC⋅cm^−2^, compared to IrO_x_ coating [100]. However, EDOT has low solubility in water and PEDOT has poor mechanical adhesion on the electrode because of its brittle structure [101,102]. Dopant can be introduced to improve the structural and properties of CP coatings, for example, polystyrene sulfonate (PSS) doped PEDOT displayed aggregation on the edge of the electrode while ClO_4_^−^ doped ones showed more even distribution [103]. Furthermore, the performances of CP coatings could be improved by means of structural design and surface functionalization. Abidian et al. [104] developed a 3D nanowire modified CP coating by electrospinning, demonstrating better elasticity and more porous morphology, which could help delivery drugs conveniently (Figure 10b). Templating was also used to create porous structures, the surface area could be increased by decreasing the size of polystyrene (PS) sphere, showing even lower impedance than bare electrode (Figure 10c), whereas the harmful solvents and residues in the templates needed to be further removed [105]. In addition, bioactive molecules could be added into the monomer of CPs in order to improve its biocompatibility and decrease the corresponding tissue response. Cui et al. [106] integrated Tyr-IIe-Gly-Ser-Arg (YIGSR) peptide into CPs to obtain PPy/DCDPGYIGSR composite coating (Figure 10d) by electrodeposition. Their results showed that neural cells grew on 83% of the electrode sites with integrated peptides while only 10% on that without peptides. Richardson et al. [107] developed PPy/pTS/NT3 (Neurotrophin-3) which effectively avoided the degradation of auditory neurons and promoted the growth toward the electrode (Figure 10e). However, the long-term in vivo stability of CPs was still undesirable, which should be further studied in future since it remained a limiting factor for implantable electrodes. 

### 4.3. Carbon Materials

**Figure 11:**
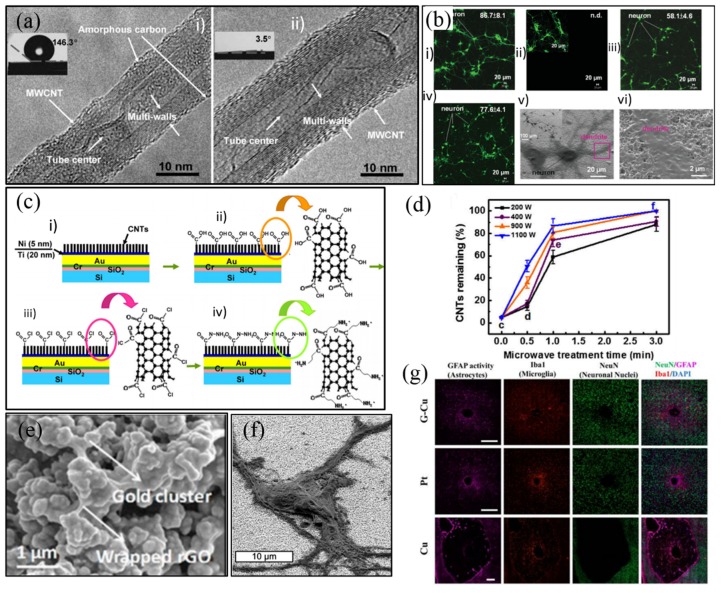
(**a**) Transmission electron microscope (TEM) of multi-walled carbon nanotube (MWCNT) with amorphous carbon before and after plasma treatment [115]; (**b**) fluorescent images of neuron cells cultured on as-grown carbon nanotubes (CNTs) and UV-ozone-modified CNTs, and corresponding SEM images [117]; (**c**) Process flow of MWCNT amino-functionalization [118]; (**d**) the percentages of CNTs remaining after 5 min sonication vs. microwave treatment time at various powers [123]; (**e**) SEM image of reduced graphene oxide (GO) coating [124]; (**f**) SEM images of neural cells attached well to GO coating [125]; (**g**) histological studies of tissue response to GO coating [122]. Reproduced from the mentioned references with permission from the related journals.

Carbon materials are considered as another promising candidate since their toxicity is minimal compared to metallic materials [110]. Among them, carbon nanotube (CNT) shows the biggest advantage as neural electrode coating because of their high surface-to-volume ratio [111] and capacitive interfacial behavior during charge transfer [112]. Although the CIC of CNT was lower than that of IrO_x_, it still showed safe stimulation in vitro [113]. CNT-coated electrode was found to be more sensitive to cell response than Pt electrode in vitro [114]. The properties of CNT was determined by its fabrication method. Chemical vapor deposition (CVD) was one of the most widely used technique, but the high temperature manufacturing process constrained the choice of electrode and substrate materials and needed additional transfer processes to polymer substrates [115]. Plasma treatment [115] and heat treatment [116] were adopted to remove amorphous carbon formed on the surface of CNTs during CVD process, which helped increasing 2-fold in capacitance but reduced the mechanical stability [116]. Furthermore, its high hydrophobicity significantly limited charge injection at the interface [113]. Thus, plasma treatment could be applied to form hydrophilic chemical bonds such as C–OH, C=O and OH–C=O, and the water contact angle could be also decreased by ultraviolet (UV)-ozone treatment (~145°) as shown in Figure 11a [115], leading to the increase of capacitance up to 80 folds. It could also promote the attachment and differentiation of neural cells to some degree *in vitro*, shown in Figure 11b [117]. Meanwhile, amination could further improve the electrochemical performance of CNT (Figure 11c) [118]. Almost no detachment occurred on the substrate after sonication, showing great adhesion strength (Figure 11d) [118]. However, their potential biotoxicity was still the main disadvantage.

In contrast, graphene bypassed this problem due to its planar geometry as well as good biocompatibility, leading to enhanced adhesion and viability [119]. Electrophoresis was usually employed to obtain graphene oxide (GO, Figure 11e) which exhibited high conductivity of 2000 S/m and low impedance, however, its smooth morphology limited its CSC_c_ lower than CNT [120,121]. The GO coating exhibited good cell viability more than 90% (Figure 11f), and it could reduce tissue responses with microglia and astrocytes distribution scattered around the coating in vitro study (Figure 11g) [122].

### 4.4. Composite Coatings

**Figure 12:**
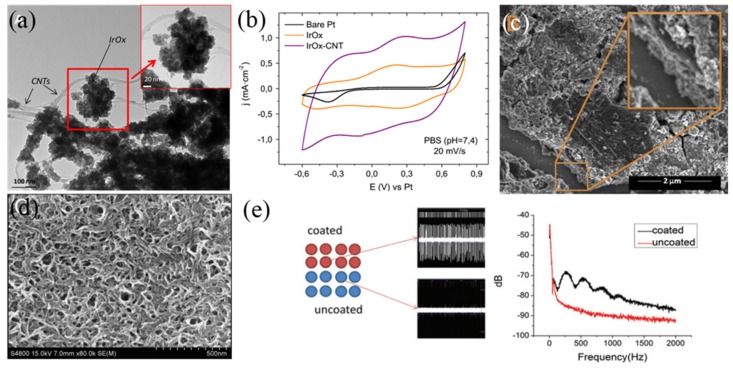
(**a**) TEM image of IrO_x_-CNT composite coating [127]; (**b**) Cyclic voltammetry (CV) curves of bare Pt, IrO_x_, and IrO_x_-CNT coated electrodes in PBS (pH = 7.4) at sweep rate of 20 mV⋅s^−1^ [127]; (**c**) SEM image of IrO_x_-GO composite coating [129]; (**d**) SEM image of SWNT-PPy composite coating [130]; (**e**) Influence of peptide bond on PEDOT/PSS/MWCNT composite coating [131]. Reproduced from the mentioned references with permission from the related journals.

Although different materials have been studied for neural interfaces, no ideal material can perform well on all properties including electrical, mechanical, and biological. Hence, composites have been recently considered an attractive option to take advantages of several materials and bypass their disadvantages. Ferguson et al. [126] fabricated Au-CNT composite coating by combining the advantages of both materials, the mechanical adhesion was inherently improved as well as its surface area. The impedance of Au-CNT could be reduced by at least 10 times with much higher CSC_c_ than that of activated iridium oxide film (AIROF), while the CIC was less than 1 mC⋅cm^−2^, since only a small amount of Faraday charge transferred between the tissue–electrode interface [126]. IrO_x_ could be used as an encapsulation layer for carbon bracket, which not only promoted more charge exchanging but also prevented carbon releasing [127]. Connecting IrO_x_ to CNT through the carboxylic acid groups improved the strength between them, leading to high effective surface area (Figure 12a) and much higher CSC_c_ of 101.2 mC⋅cm^−2^ than pure IrO_x_ (Figure 12b) [127]. Moreover, IrO_x_ could be combined with reduced graphene oxide (RGO) or GO to form composite coatings, among which IrO_x_-GO showed larger CSC_c_ because of its rougher surface (Figure 12c) and remained more than 10% higher CSC_c_ than that of pure IrO_x_ and IrO_x_-CNT after 1000 CV cycles, demonstrating great electrochemical stability [128,129].

In addition, combining CPs with nanostructured carbon materials (NC) like CNTs may also provide a highly electroactive, mechanically strong, and biocompatible coating. Zhou et al. [132] doped multi-walled CNT (MWCNT) in PEDOT to get PEDOT-MWCNT composite coating, exhibiting superior stability to pure PEDOT coating with only 2% loss of CSC_c_ after electrochemical stimulating for 96 h under 3 mC⋅cm^−2^ pulse. Compared to co-deposition of CP-NC coatings, electrodeposited CPs on NC layer by layer was more controllable (Figure 12d), the 3D independent topography could provide rougher surface to transfer charge with more electroactive points [133]. Hydrogels, biomolecules, etc. were often incorporated into the coating to further improve the biocompatibility, which was shown to benefit in vivo recording supported by more active channels and higher signal power than pure coating (Figure 12e) [131]. Anti-inflammatory drugs could also be incorporated in those composite coatings for alleviation [134].

## 5. Conclusions

This review summarizes the micro/nano technologies for high-density implantable packaging and fMEA as well as high-performance coating materials in the retinal prosthesis, which are crucial to achieve high resolution. Implantable biomedical component encapsulation is important, and high density, air tightness and biocompatible electronic packaging is an inevitable choice to build a sealed cavity with mechanical support performance and biological compatibility. Also, high-density microelectrode arrays should be capable of precise stimulation, while high-performance coating materials would help improve the implanted electrodes equipped with high CSC_c_ and CIC, and corresponding low impedance to ensure safe stimulation efficiently. It is worth considering that the chronic encapsulation, high-density fMEA together with high-performance coating materials contribute to high-density retinal implants, which lead to higher resolution, however, such hardware improvements need to be combined with better stimulation strategies and the latest progress in neuroscience and ophthalmology to realize the precise, effective, and chronic artificial vision.

Although the performance of retinal implants has been significantly improved through development in the past few decades, the design of the overall components is far from perfect, especially for high-density implants. There are still great challenges in high-density fabrication and integration, low invasiveness, power consumption, and high biocompatibility in the future. New concepts and materials are being introduced in the research of artificial vision, such as optogenetics [135], gene therapy [136], flexible photovoltaic films [137], and nanowire [138]. Future research efforts should target a more solid advancement in electronics, microfabrication, material science, and new biotechnologies in order to better understand retinal processing, so that the retinal implant will be easily accepted by the public with low cost.
